# Antibacterial activity and cytocompatibility of Cu-coated PETG for clear aligner applications

**DOI:** 10.3389/fbioe.2026.1838596

**Published:** 2026-06-26

**Authors:** Zongfei Chen, Sihang Chen, Pengcheng Huang, Jingjing Su, Linyu Xu

**Affiliations:** 1 Fujian Key Laboratory of Oral Diseases & Fujian Provincial Engineering Research Center of Oral Biomaterial & Stomatological Key Lab of Fujian College and University, School and Hospital of Stomatology, Fujian Medical University, Fuzhou, China; 2 Stomatological Hospital of Xiamen Medical College, Xiamen Key Laboratory of Stomatological Disease Diagnosis and Treatment, Xiamen, China

**Keywords:** antibacterial activity, biofilm inhibition, clear aligner, copper coating, PETG

## Abstract

Prolonged wear of clear aligners may promote plaque accumulation and increase the risks of enamel demineralization and gingival inflammation. In this study, a copper (Cu) coating was fabricated on polyethylene terephthalate glycol (PETG) by magnetron sputtering, and its physicochemical properties, antibacterial activity, antibiofilm effect, and cytocompatibility were evaluated. Surface characteristics were analyzed by SEM, EDS, AFM, XRD, and FTIR, and mechanical properties were also assessed. Antibacterial and antibiofilm activities against *Streptococcus mutans* and *Porphyromonas gingivalis* were evaluated by CFU counting, MTT assay, and CLSM, while cytocompatibility was assessed by CCK-8 assay and cytoskeletal staining. The results showed that a relatively uniform Cu coating was successfully formed on the PETG surface without markedly affecting the main chemical structure or mechanical properties of PETG. Compared with pristine PETG, PETG/Cu composites exhibited evident antibacterial effects against both bacterial species, with the 45 s group showing the best performance, reaching antibacterial rates of approximately 60% against *S. mutans* and 80% against *P. gingivalis,* while also reducing bacterial adhesion and biofilm formation. No apparent cytotoxicity was observed under the tested *in vitro* conditions. These findings indicate that PETG/Cu composite coatings can endow PETG with antibacterial and antibiofilm properties while maintaining its basic performance, highlighting its potential for functional clear aligner applications.

## 1 Introduction

Clear aligner therapy has become an important modality in contemporary orthodontic treatment because of its favorable esthetics, comfort, and removability. Polyethylene terephthalate glycol-1,4-cyclohexanedimethanol (PETG), characterized by high transparency, favorable mechanical properties, and good biocompatibility, has been widely used as a major substrate material for clear aligners (Ravuri et al., 2024; Bichu et al., 2023). However, because aligners are typically worn for prolonged periods, the tooth surface remains in a relatively enclosed microenvironment beneath the appliance, which facilitates the accumulation of food debris and dental plaque, promotes the colonization and proliferation of oral pathogenic microorganisms, and further increases the risks of enamel demineralization, white spot lesion formation, and gingival inflammation (Levrini et al., 2015; Xia et al., 2025).

Oral health during aligner treatment is also closely associated with the surface properties of the aligner material (Martínez Gil-Ortega et al., 2025; Yalçın and Kavasoğlu, 2025). PETG surfaces are relatively hydrophobic and intrinsically lack active antibacterial properties, characteristics that may facilitate early bacterial adhesion and subsequent biofilm formation. A recent study showed that PETG wear could decrease salivary pH from 7.54 to 7.14 within 4 h (*P* < 0.05), thereby promoting the metabolism of cariogenic bacteria and increasing the relative abundance of *Streptococcus* to 40.7% at approximately 8 h (Gao et al., 2025). In addition, enrichment of anaerobic pathogens such as *Porphyromonas gingivalis* may occur in regions where the aligner contacts the gingival tissues, thereby contributing to periodontal inflammatory responses (Zhang et al., 2020).

At present, plaque control associated with clear aligner therapy still mainly depends on daily cleaning and maintenance by patients, and its practical effectiveness is highly influenced by patient compliance (Pardo et al., 2024). To endow aligner materials with active antibacterial properties, previous studies have explored strategies such as incorporating antibacterial agents into the matrix (Alqarni et al., 2025) or constructing functional antibacterial coatings on the material surface (Wang et al., 2023). The former is relatively straightforward, but the distribution, exposure, and sustained activity of the antibacterial components may limit its long-term efficacy. In contrast, surface functionalization can more directly regulate the biological behavior of the material interface, although its practical application still requires balancing antibacterial durability, biocompatibility, preservation of appearance, and manufacturing complexity. Therefore, the development of a relatively simple, stable, and PETG-compatible antibacterial modification strategy remains of considerable research interest.

Among various antibacterial materials, metal-based materials have attracted extensive attention because of their unique physicochemical properties and broad-spectrum antimicrobial potential (Pahlevanzadeh et al., 2022). Copper-based materials have shown promising prospects in oral biomaterials because of their relatively low cost, high antibacterial efficiency, and low tendency to induce bacterial resistance (Ma et al., 2022). Previous studies have shown that copper and its oxides exert antibacterial effects through multiple mechanisms, including copper ion release, reactive oxygen species generation, disruption of bacterial membrane integrity, and interference with nucleic acid and protein metabolism (Wu et al., 2026), while also inhibiting bacterial biofilm formation to a certain extent. Therefore, introducing copper onto the PETG surface to construct a functional antibacterial coating may improve the antibacterial and antibiofilm properties of clear aligner materials and provide a new materials-based strategy for reducing plaque accumulation and the associated risks of caries and gingival inflammation during aligner treatment.

Magnetron sputtering, a commonly used physical vapor deposition (PVD) technique, enables the controllable deposition of thin films under vacuum conditions and offers several advantages, including uniform film formation, tunable coating thickness and composition, good process reproducibility, and a relatively clean fabrication process (Borowski and Myśliwiec, 2025). More importantly, this technique mainly acts on the material surface, making it possible to introduce new surface functions while minimizing changes to the bulk properties of the substrate (Santo et al., 2025). For polymeric substrates such as PETG, magnetron sputtering also features relatively low thermal impact and suitability for low-temperature deposition (Garg et al., 2024), suggesting its potential advantages for constructing functional antibacterial coatings on PETG surfaces.

Based on these considerations, the present study aimed to construct a Cu coating on PETG by magnetron sputtering while preserving the basic properties of the PETG substrate as much as possible. The surface morphology, structural characteristics, mechanical properties, antibacterial and antibiofilm performance, and cytocompatibility of the modified materials were systematically evaluated, in order to provide an experimental basis for the surface functionalization and potential application of clear aligner materials.

## 2 Materials and methods

### 2.1 Preparation of PETG/Cu composites

Polyethylene terephthalate glycol (PETG) sheets (Keystone Industries, United States) were used as the substrate material. For surface characterization and biological assays, the PETG sheets were cut into specimens measuring 10 mm × 10 mm, sequentially ultrasonically cleaned in acetone, absolute ethanol, and deionized water for 15 min each, and then air-dried. The dried samples were subjected to bilateral surface treatment using a plasma cleaner (Harrick Plasma, United States) and stored in a vacuum drying oven before further use.

Cu deposition was performed on one side of the PETG samples using a magnetron sputtering system (nanoPVD, Moorfield, United Kingdom) with a high-purity copper target (99.99%; Sigma-Aldrich, United States) as the sputtering source. Subsequent surface characterization and biological assays were performed on the Cu-coated side. According to the sputtering duration, the samples were divided into four groups: pristine PETG (0 s), PETG/Cu-15 s, PETG/Cu-30 s, and PETG/Cu-45 s. Before antibacterial and cytocompatibility assays, all samples were immersed in 75% ethanol for 30 min, rinsed three times with sterile PBS, and then exposed to ultraviolet irradiation for 30 min on each side for sterilization.

### 2.2 Material characterization

The crystal structure of the samples was analyzed by X-ray diffraction (XRD; Philips, Netherlands). Surface morphology and microstructure were observed using scanning electron microscopy (SEM; CIQTEK, China), and elemental composition was analyzed by energy-dispersive X-ray spectroscopy (EDS) coupled with SEM. Surface nanoscale topography was examined using atomic force microscopy (AFM; Nano Wizard 4, Bruker, Germany). Surface chemical structures were analyzed by Fourier transform infrared spectroscopy (FTIR; Cary 660 FTIR, Agilent, United States) equipped with an attenuated total reflectance (ATR) accessory.

### 2.3 Mechanical testing

The mechanical properties of the samples were evaluated using a universal electronic testing machine (Sans, China). For tensile testing, the PETG sheets were cut into specimens with a gauge length of 20 mm, a gauge width of 10 mm, and a thickness of 1.05 mm. The crosshead speed was set at 15 mm/min. Stress-strain curves were recorded during tensile testing, and tensile strength, elastic modulus, maximum force, and elongation at break were analyzed to assess the effect of Cu deposition on the mechanical performance of the PETG substrate. Three independent specimens were tested for each group.

### 2.4 Evaluation of antibacterial properties

#### 2.4.1 Bacterial strains and culture conditions

*Streptococcus mutans* (UA159) and *Porphyromonas gingivalis* (ATCC 33277) were used as the test strains. *S. mutans* was cultured in brain heart infusion (BHI) medium (Solarbio, China) at 37 °C under microaerophilic conditions, whereas *P. gingivalis* was cultured in BHI medium supplemented with hemin and vitamin K (Sigma-Aldrich, United States) at 37 °C under anaerobic conditions. After revival and subculture, bacterial suspensions were adjusted to approximately 1 × 10^6^ CFU/mL for subsequent experiments.

#### 2.4.2 Colony-forming unit counting assay

The antibacterial activity of the sample surfaces was evaluated with reference to the principles of ISO 22196. Sterilized samples from each group were placed in sterile culture dishes, and 0.4 mL of bacterial suspension (approximately 1 × 10^6^ CFU/mL) was added onto each sample. The inoculum was then covered with a sterile polypropylene (PP) film to ensure even distribution across the surface. The samples were incubated for 24 h under the corresponding culture conditions.

After incubation, 1 mL of sterile phosphate-buffered saline (PBS, pH 7.4) was added as the elution solution, and the samples were thoroughly pipetted and vortexed to recover adherent bacteria from the surfaces. The recovered bacterial suspensions were serially diluted, plated onto the corresponding agar media, and cultured for colony-forming unit (CFU) counting. Five replicates were included in each group. Colony counts were further analyzed using ImageJ software.

#### 2.4.3 Bacterial metabolic activity assay

The metabolic activity of bacteria adhering to the PETG/Cu composite surfaces was evaluated using a 3-(4,5-dimethylthiazol-2-yl)-2,5-diphenyltetrazolium bromide (MTT) assay. Sterilized samples (1 cm × 1 cm) from each group were placed in 24-well plates, and 1 mL of bacterial suspension (1 × 10^6^ CFU/mL) was added to each well. After co-culture for 24 h under the corresponding conditions, the culture medium was carefully removed, and the samples were gently rinsed with sterile PBS to remove non-adherent bacteria. Fresh medium containing MTT solution was then added to each well to achieve a final MTT concentration of 0.5 mg/mL. The plates were incubated at 37 °C for 2 h in the dark. After removal of the MTT-containing solution, dimethyl sulfoxide (DMSO) was added to dissolve the formazan crystals. The dissolved solution was transferred to a 96-well plate, and the absorbance was measured at 540 nm using a microplate reader (Spectra iD3, Molecular Devices, United States). The absorbance value was used to reflect the metabolic activity of bacteria adhering to the material surface.

#### 2.4.4 Live/dead bacterial staining and confocal observation

Bacterial viability on the sample surfaces was evaluated using a LIVE/DEAD bacterial viability kit (Beyotime Biotechnology, China). After co-culture with the different samples for 24 h, the samples were gently rinsed with sterile PBS to remove non-adherent bacteria and then stained according to the manufacturer’s instructions. After incubation in the dark for 15 min, the stained samples were directly observed by confocal laser scanning microscopy (CLSM). Green fluorescence indicated live bacteria, whereas red fluorescence indicated dead bacteria.

### 2.5 Evaluation of cytocompatibility

#### 2.5.1 Cell culture

Human oral keratinocytes (HOKs; #2610, ScienCell, United States) were cultured in Dulbecco’s modified Eagle medium (DMEM) supplemented with 10% fetal bovine serum (FBS; Baobio™ ([300,501], Hangzhou, China) in a humidified incubator at 37 °C with 5% CO_2_.

#### 2.5.2 CCK-8 cell viability assay

Samples from each group were cut into 2.4 cm × 2.4 cm pieces and immersed in 4 mL of DMEM, corresponding to an approximate extraction ratio of 3 cm^2^/mL, for 3 days at 37 °C in 5% CO_2_ to prepare material extracts. HOKs were seeded into 96-well plates at a density of 2 × 10^3^ cells per well. After attachment and growth to approximately 70% confluence, the original culture medium was replaced with 100 μL of the corresponding extract in each well. Cells cultured in complete medium served as the negative control. After incubation for 24, 48, or 72 h, 10 μL of Cell Counting Kit-8 (CCK-8; Dojindo Laboratories, Japan) reagent was added to each well, followed by an additional 2 h incubation. Absorbance at 450 nm was measured using a microplate reader (Spectra iD3, Molecular Devices, United States). Five replicates were included in each group.

#### 2.5.3 Cytoskeletal staining

HOKs were seeded into confocal dishes (Biosharp, China) at a density of 8 × 10^4^ cells/dish. After cell attachment, the culture medium was replaced with extracts from the different material groups. For the 5-day culture, the extracts were refreshed every 2 days. At days 1 and 5, the cells were washed three times with phosphate-buffered saline (PBS), fixed with 4% paraformaldehyde for 20 min, and permeabilized with 0.4% Triton X-100 for 10 min. After another PBS wash, the cells were stained with rhodamine-conjugated phalloidin (Uelandy, China) for 20 min in the dark and counterstained with DAPI (Thermo Fisher Scientific, United States) for 5 min at room temperature. After washing with PBS, the cytoskeleton and nuclei were observed using a confocal fluorescence microscope.

### 2.6 Statistical analysis

All data are presented as mean ± standard deviation (mean ± SD). Image analysis was performed using ImageJ software, and statistical analyses were conducted using GraphPad Prism 10 software. Comparisons among groups were performed using one-way analysis of variance (one-way ANOVA) followed by Tukey’s post hoc test. A value of *P* < 0.05 was considered statistically significant.

## 3 Results

### 3.1 Surface characterization, chemical structure, and mechanical properties of PETG/Cu composites

**FIGURE 1:**
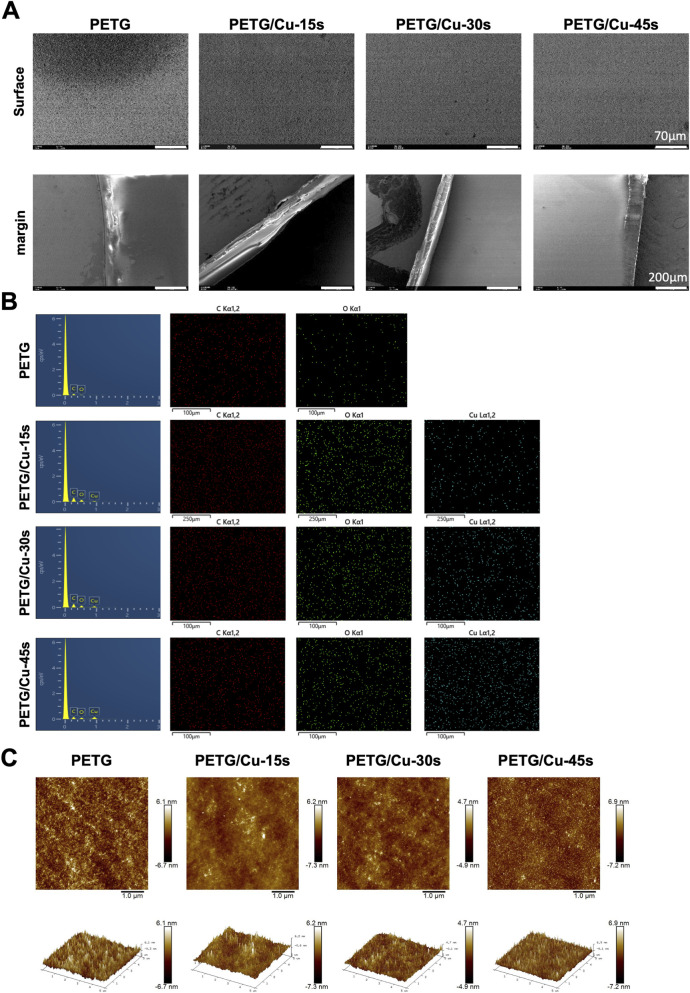
Surface and morphological characterization of PETG/Cu composites prepared with different deposition durations. **(A)** Scanning electron microscopy (SEM) images of the sample surfaces. Scale bars: 70 μm (upper row) and 120 μm (lower row). **(B)** Energy-dispersive X-ray spectroscopy (EDS) analysis. The Cu content increased from 0 at% in pristine PETG to 1.12 at%, 2.62 at%, and 6.20 at% in the 15 s, 30 s, and 45 s groups, respectively. **(C)** Atomic force microscopy (AFM) images of the different samples.

**FIGURE 2:**
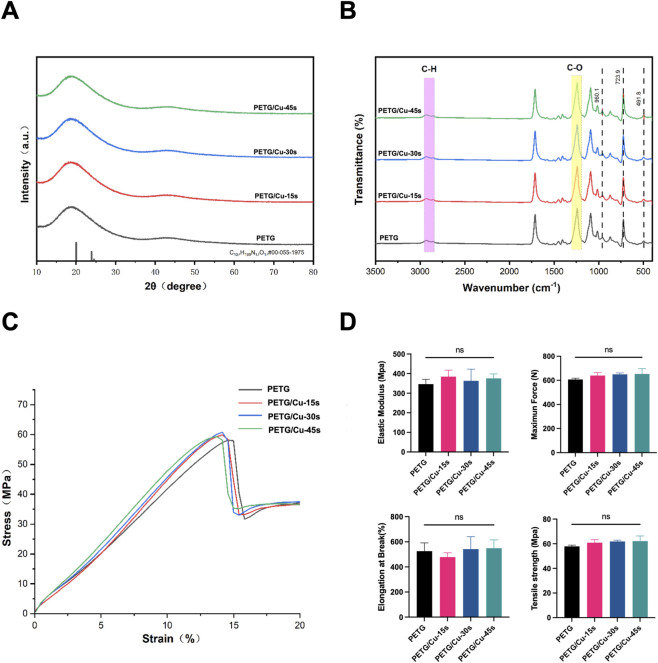
XRD, FTIR, and mechanical characterization of PETG/Cu composites prepared with different deposition times. **(A)** XRD patterns. The reference pattern shown at the bottom corresponds to PETG, the polymeric substrate, and is included only for comparison purposes. It should not be interpreted as a crystalline metallic Cu reference pattern or as direct evidence for crystalline Cu formation. **(B)** FTIR spectra. **(C)** Stress-strain curves. **(D)** Quantitative analysis of the mechanical properties, including elongation at break, elastic modulus, maximum force, and tensile strength. No significant differences were observed among groups (*P* > 0.05; ns, not significant).

Representative surface characterization and mechanical testing results of pristine PETG and PETG/Cu composites prepared with different Cu deposition durations (15, 30, and 45 s) are shown in Figures 1, 2.

SEM images showed that the surface of pristine PETG was relatively smooth, with no obvious particulate structures observed (Figure 1A). After Cu sputtering, the PETG/Cu groups showed subtle surface morphological changes compared with pristine PETG. Slightly roughened or particulate-like surface features were observed locally, particularly in the marginal regions, and these features appeared relatively more evident in the 45 s group.

EDS analysis provided elemental evidence for the introduction of Cu onto the PETG surface (Figure 1B). Only C and O signals were detected on the surface of pristine PETG, whereas Cu signals were observed in all PETG/Cu groups. Moreover, the Cu signals in the 30 s and 45 s groups were more evident than those in the 15 s group. Semi-quantitative EDS analysis further showed that the surface Cu content increased with sputtering time, reaching approximately 1.12 at%, 2.62 at%, and 6.20 at% in the 15 s, 30 s, and 45 s groups, respectively, indicating that the surface Cu content increased with prolonged sputtering time.

AFM analysis showed that the overall surface fluctuations of all groups remained relatively small, suggesting that the PETG substrate largely retained favorable surface flatness after treatment (Figure 1C). Quantitative roughness analysis further showed that the Ra values of pristine PETG, PETG/Cu-15 s, PETG/Cu-30 s, and PETG/Cu-45 s were 1.11, 0.668, 0.529, and 0.900 nm, respectively, while the corresponding Rq values were 1.43, 0.913, 0.722, and 1.21 nm, respectively. These results indicate that although SEM revealed localized marginal surface changes after sputtering, the overall nanoscale roughness of the PETG/Cu groups remained low and did not exceed that of pristine PETG.

XRD analysis showed that pristine PETG and PETG/Cu composites exhibited similar diffraction profiles characterized by a broad amorphous background of the PETG substrate (Figure 2A). No sharp or well-resolved diffraction peaks attributable to crystalline metallic Cu or copper oxides were clearly identified in the PETG/Cu groups. These results suggest that Cu sputtering did not induce detectable crystalline phase changes in the PETG substrate under the present XRD conditions. Therefore, the XRD results alone were insufficient to confirm the formation of crystalline Cu or copper oxide phases. In the present study, the presence of Cu on the PETG surface and the sputtering-time-dependent increase in surface Cu content were mainly supported by the EDS results.

FTIR analysis showed that all PETG/Cu composites retained the main characteristic absorption peaks of the PETG substrate, with the strongest peak located near 1,239 cm^-1^ (Figure 2B), suggesting that the magnetron sputtering process did not markedly alter the main chemical structure of the PETG matrix. In addition, a weak absorption band was observed near 420 cm^-1^, and its intensity slightly increased with prolonged Cu sputtering time. This change may be associated with Cu-related surface species and was generally consistent with the EDS results.

Mechanical testing results showed that the overall trends of the stress-strain curves were similar among pristine PETG and all PETG/Cu groups, with peak stresses ranging from 60 to 70 MPa (Figures 2C,D). Further analysis of elongation at break, elastic modulus, maximum force, and tensile strength revealed no statistically significant differences among the groups (*P* > 0.05), indicating that Cu sputtering did not markedly affect the mechanical properties of PETG.

Collectively, these results demonstrated that magnetron sputtering enabled Cu functionalization of the PETG surface, with increased surface Cu content observed at longer sputtering times, while the main chemical structure and mechanical properties of the PETG substrate remained largely unchanged.

### 3.2 Antibacterial and antibiofilm activities of PETG/Cu composites against *Streptococcus mutans* increased with Cu deposition time

**FIGURE 3:**
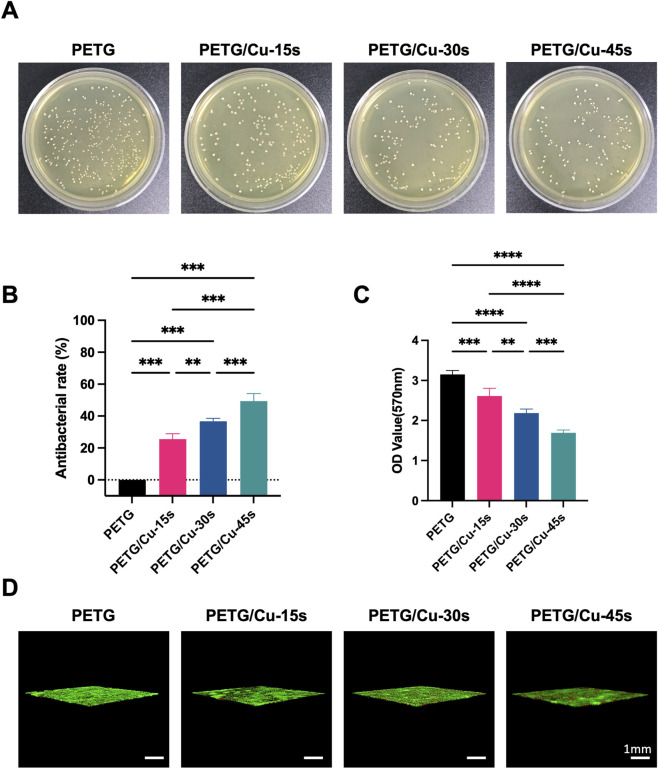
Antibacterial and antibiofilm activities of PETG surfaces with different Cu deposition times against *Streptococcus mutans*. **(A)** Representative colony images after co-culture with *S. mutans*. **(B)** Quantification of the antibacterial rate. **(C)** MTT assay of bacterial metabolic activity after 24 h of co-culture. **(D)** CLSM images of live/dead-stained *S. mutans* biofilms on the sample surfaces. Scale bar = 1 mm **P < 0.01, ***P < 0.001, ****P < 0.0001.

The antibacterial and antibiofilm activities of pristine PETG and PETG/Cu composites prepared with different Cu deposition durations against *Streptococcus mutans* (*S. mutans*) are summarized in Figure 3.

CFU counting showed that a large number of dense colonies were observed in the pristine PETG group, whereas the number of colonies was reduced in all PETG/Cu groups (Figure 3A). Moreover, the colony number gradually decreased with increasing Cu sputtering time, with the 45 s group showing the fewest colonies, indicating the strongest inhibitory effect on viable *S. mutans* colonization on the material surface.

The antibacterial rate analysis yielded results consistent with the CFU data (Figure 3B). Compared with pristine PETG, all PETG/Cu groups exhibited antibacterial activity, and the antibacterial rate increased with prolonged Cu deposition time. Among all groups, the 45 s group showed the highest antibacterial rate, reaching approximately 60%.

MTT assay results further supported these findings (Figure 3C). The pristine PETG group showed the highest absorbance value, whereas all PETG/Cu groups exhibited lower absorbance values, which gradually decreased with increasing deposition time. The lowest absorbance value was observed in the 45 s group, suggesting that Cu coating effectively inhibited the metabolic activity of *S. mutans* adhering to the material surface.

CLSM observations showed that many live bacteria with green fluorescence were present on the pristine PETG surface, forming a relatively dense biofilm structure (Figure 3D). In contrast, all PETG/Cu groups exhibited markedly reduced bacterial adhesion and weakened biofilm structures, and this trend became more evident with increasing Cu deposition time. The strongest inhibitory effect was observed in the 45 s group.

Collectively, the CFU counting, antibacterial rate analysis, MTT assay, and CLSM observations consistently demonstrated that Cu coating significantly enhanced the antibacterial and antibiofilm activities of PETG against *S. mutans*, and this effect increased with prolonged Cu deposition time.

### 3.3 Antibacterial and antibiofilm activities of PETG/Cu composites against *Porphyromonas gingivalis* increased with Cu deposition time

**FIGURE 4:**
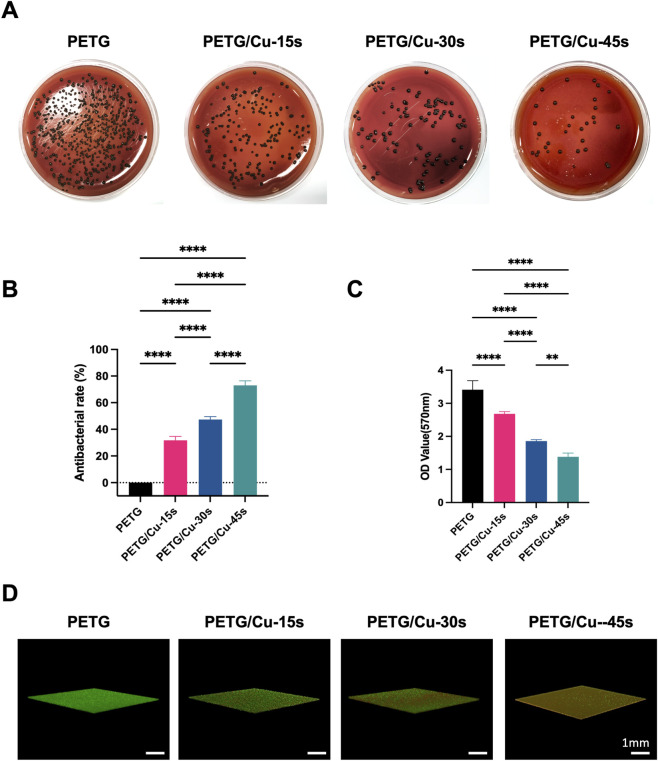
Antibacterial and antibiofilm activities of PETG surfaces with different Cu deposition times against *Porphyromonas gingivalis*. **(A)** Representative colony images after co-culture with *P. gingivalis*. **(B)** Quantification of the antibacterial rate. **(C)** MTT assay of bacterial metabolic activity after 24 h of co-culture. **(D)** CLSM images of live/dead-stained *P. gingivalis* biofilms on the sample surfaces. Scale bar = 1 mm **P < 0.01, ****P < 0.0001.

The antibacterial and antibiofilm activities of pristine PETG and PETG/Cu composites prepared with different Cu deposition durations against *Porphyromonas gingivalis* (*P. gingivalis*) are summarized in Figure 4.

CFU counting showed that a large number of dense colonies were observed in the pristine PETG group, whereas the number of colonies was markedly reduced in all PETG/Cu groups (Figure 4A). Moreover, the colony number further decreased with increasing Cu sputtering time. Only a few sparse colonies were observed in the 45 s group, indicating the most pronounced surface antibacterial effect against *P. gingivalis*.

The antibacterial rate analysis showed that the antibacterial rates of the 15 s, 30 s, and 45 s groups gradually increased to approximately 30%, 50%, and 80%, respectively, compared with pristine PETG (Figure 4B). These results indicated that PETG/Cu composites exerted evident inhibitory effects on *P. gingivalis*, and that the antibacterial activity increased with prolonged deposition time.

MTT assay results showed that the pristine PETG group exhibited the highest absorbance value, whereas the absorbance values of the PETG/Cu groups gradually decreased with increasing deposition time, with the lowest value observed in the 45 s group (Figure 4C), suggesting the strongest inhibitory effect on the metabolic activity of *P. gingivalis* on the material surface.

CLSM observations showed strong green fluorescence on the pristine PETG surface, indicating a higher level of viable bacterial attachment (Figure 4D). In contrast, the fluorescence signals on the PETG/Cu groups were markedly weakened, and the bacterial adhesion density further decreased with increasing Cu deposition time. The strongest inhibitory effect was observed in the 45 s group.

Collectively, the CFU counting, antibacterial rate analysis, MTT assay and CLSM observations consistently demonstrated that PETG/Cu composites exhibited evident antibacterial and antibiofilm activities against *P. gingivalis*, and that this effect was enhanced with prolonged Cu deposition time.

### 3.4 Cytocompatibility of PETG/Cu composites

**FIGURE 5:**
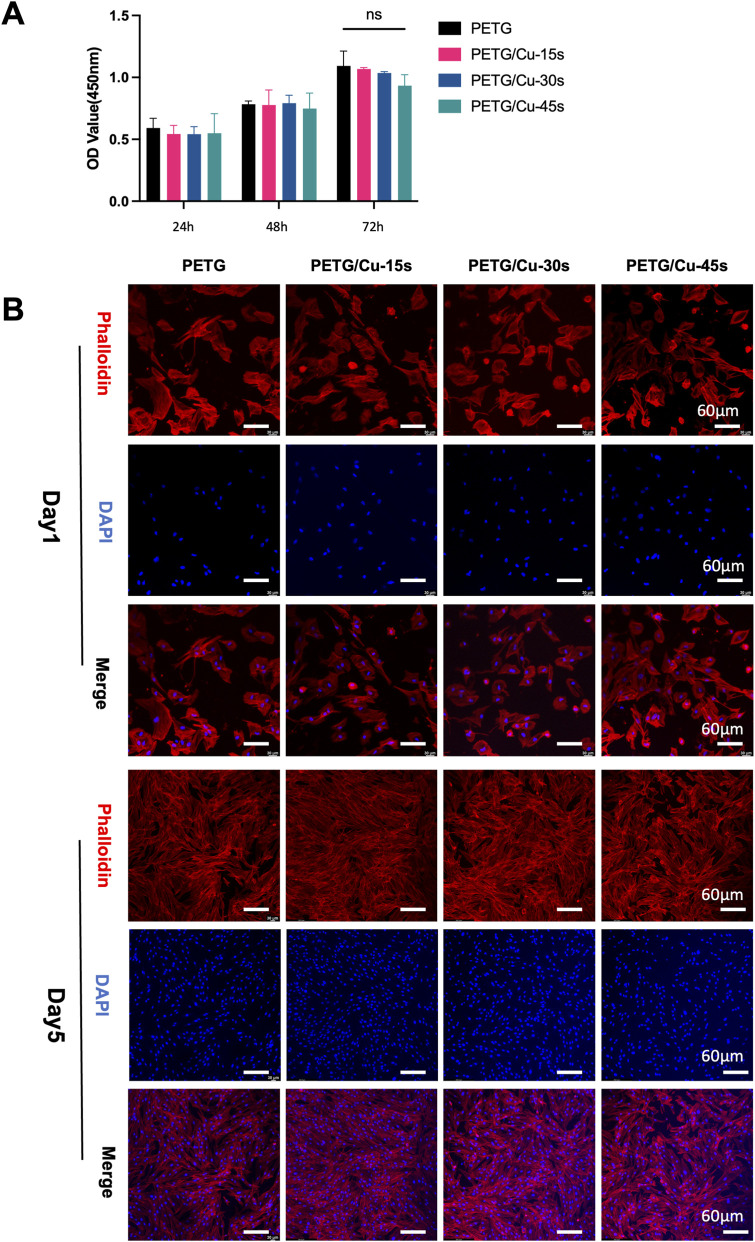
Cytocompatibility of PETG/Cu composites prepared with different Cu deposition times. **(A)** Cell viability at 24, 48, and 72 h (ns, not significant). **(B)** Cytoskeletal staining images on day 1 and day 5. Red fluorescence indicates the cytoskeleton, and blue fluorescence indicates the nuclei. Scale bar = 60 μm.

The effects of pristine PETG and PETG/Cu composites prepared with different Cu deposition durations (15, 30, and 45 s) on cell viability and morphology were evaluated by Cell Counting Kit-8 (CCK-8) assay and confocal laser scanning microscopy (CLSM). The results are shown in Figure 5.

The CCK-8 results showed that after 24, 48, and 72 h of culture, no statistically significant differences in cell viability were observed between the PETG/Cu groups and the pristine PETG group (Figure 5A, *P* > 0.05), indicating that PETG/Cu composites prepared with different Cu deposition durations did not exert obvious adverse effects on cell viability.

CLSM observations further showed that cells in all groups exhibited favorable adhesion and spreading under both short-term (D1) and long-term (D5) culture conditions (Figure 5B). Phalloidin staining revealed intact actin filaments, well-spread cell morphology, and uniformly distributed nuclei in all groups, with no obvious cell shrinkage, poor attachment, or abnormal morphology observed.

Collectively, the CCK-8 and CLSM results demonstrated that PETG/Cu composites prepared with different Cu deposition durations exhibited good cytocompatibility and did not show obvious cytotoxicity.

## 4 Discussion

Clear aligners have been widely used in orthodontic treatment because of their favorable esthetics and comfort. However, prolonged wear may also alter the local microenvironment on the tooth surface and increase the risks of plaque accumulation, enamel demineralization, and gingival inflammation. Therefore, endowing PETG-based aligner materials with surface antibacterial functionality while preserving their original properties has become an important direction for material modification. In the present study, Cu functionalization of the PETG surface was achieved by magnetron sputtering, and its surface characteristics, antibacterial and antibiofilm performance, mechanical properties, and cytocompatibility were systematically evaluated. The results showed that this modification strategy enhanced the antibacterial and antibiofilm properties of the material without markedly affecting the main physicochemical properties of the PETG substrate, while maintaining good cytocompatibility, suggesting its potential application in the surface functionalization of clear aligner materials. From a process-optimization perspective, the 45 s group exhibited the most favorable overall performance among the tested sputtering durations. Although further prolonging the sputtering time, such as to 60 s or 90 s, may theoretically increase surface Cu loading and potentially enhance antibacterial activity, the clinical applicability of clear aligner materials cannot be evaluated solely based on antibacterial efficiency. Optical transparency, esthetic appearance, flexibility, coating stability, and cytocompatibility are essential requirements for clear aligner materials (Bichu et al., 2023). Excessive Cu deposition may potentially compromise these properties, for example, by affecting optical appearance, increasing coating stress on the flexible PETG substrate, or increasing the risk of excessive Cu ion release. In addition, a thicker metallic coating on a flexible polymer substrate may increase internal stress and the risk of microcracking or delamination during deformation. Moreover, because the biological effects of copper ions are concentration-dependent, excessive Cu loading may increase the possibility of burst Cu ion release and potential cytotoxicity (Liu et al., 2025). Therefore, within the tested parameter range in the present study, 45 s was considered to provide a practical balance between antibacterial efficacy, antibiofilm performance, material stability, and biological safety for clear aligner surface functionalization.

From the perspective of material characterization, XRD was mainly used to evaluate whether Cu sputtering induced detectable crystalline phase changes in the PETG substrate. The XRD patterns of pristine PETG and PETG/Cu composites were mainly characterized by a broad amorphous background of PETG, indicating that the overall structural features of the PETG substrate were largely preserved after sputtering. No sharp or well-resolved diffraction reflections attributable to crystalline metallic Cu or copper oxides were clearly identified in the PETG/Cu groups. This may be related to the relatively low Cu content, thin surface coating, possible limited crystallinity of the deposited layer, or masking by the amorphous PETG background. Therefore, in the present study, XRD mainly suggested that Cu sputtering did not induce obvious crystalline phase changes detectable by conventional XRD, whereas the introduction of Cu onto the PETG surface and its deposition-time-dependent increase were mainly supported by EDS analysis. Although SEM showed relatively subtle surface morphological changes after sputtering, AFM analysis further suggested that the overall nanoscale roughness remained low and did not exceed that of pristine PETG, despite the presence of localized surface microtopographical features after Cu modification.

This indicates that magnetron sputtering altered the surface microtopography while largely preserving surface smoothness. FTIR analysis further demonstrated that all groups retained the main characteristic absorption peaks of PETG, suggesting that the modification process did not markedly alter the main chemical structure of the PETG substrate. Previous studies have shown that magnetron sputtering can achieve relatively uniform and controllable deposition of metal functional layers on various substrates (Romani et al., 2021; Alfonso et al., 2012), and that the surface microstructure and elemental distribution of antibacterial coatings may affect their antibacterial efficiency (Shin et al., 2019). In addition, the construction of copper-based antibacterial active surfaces is also closely related to coating technology and the surface distribution of elements (Bharadishettar et al., 2021). Therefore, the deposition-time-dependent increase in surface Cu content detected by EDS, together with the preserved low nanoscale roughness and PETG chemical structure, may provide an important materials basis for the subsequent improvement in antibacterial performance. Collectively, these findings indicate that magnetron sputtering is a feasible approach for Cu functionalization of PETG surfaces, and that sputtering time is an important process parameter for regulating surface Cu loading.

The enhancement of antibacterial performance after Cu modification was one of the major findings of this study. Previous studies have shown that copper and its oxides exert antibacterial effects through multiple mechanisms, including copper ion release, reactive oxygen species (ROS) generation, disruption of bacterial membrane integrity, and interference with nucleic acid and protein metabolism (Ma et al., 2022). Some studies have further suggested that ROS-related processes may play an important role in the antibacterial activity of copper-based active surfaces (Wang et al., 2025). In the present study, PETG/Cu composites exhibited more pronounced inhibitory effects against both *S. mutans* and *P. gingivalis* than pristine PETG, and this effect increased with sputtering time. CFU counting, antibacterial rate analysis, and MTT-based metabolic activity results consistently showed that the 45 s group had the strongest antibacterial effect against both bacterial strains. CLSM observations further demonstrated reduced bacterial adhesion on the Cu-modified surfaces, and, particularly in the *S. mutans* model, a marked reduction in bacterial adhesion and biofilm-like structures. These results suggest that the Cu coating on PETG not only inhibited bacterial growth but may also have interfered with early bacterial adhesion and subsequent biofilm formation. Similar studies on the antibacterial modification of orthodontic appliance surfaces have also shown that metal nanoparticle-enriched surface layers can significantly enhance antibacterial and antibiofilm activity (Sycińska-Dziarnowska et al., 2025), which is generally consistent with our findings.

The two bacterial strains selected in this study represent two major microbiological risks commonly associated with clear aligner treatment. *S. mutans* is a typical cariogenic bacterium, and its growth, acid production, and biofilm formation are closely related to enamel demineralization (Legéňová and Bujdáková, 2015). In contrast, *P. gingivalis* is an important periodontal pathogen closely associated with gingival inflammation and periodontal tissue damage (How et al., 2016). The fact that PETG/Cu composites inhibited both types of bacteria suggests that this material may have potential value in reducing the colonization risks of both cariogenic and periodontal pathogenic bacteria on clear aligners. It should also be noted that the antibacterial rates observed in the present study, although lower than the >99% level often associated with conventional sterilization strategies, may still be clinically meaningful for clear aligner applications. Previous studies have shown that oral health outcomes during clear aligner treatment are closely related to plaque control and biofilm accumulation (Llera-Romero et al., 2023). In addition, the oral cavity is a dynamic microbial ecosystem rather than a sterile environment. Therefore, the clinical objective of aligner surface modification is not necessarily complete eradication of oral microorganisms, but rather sustained suppression of key pathogenic bacteria and reduction of biofilm burden during prolonged daily wear. In this context, continuous inhibition of major cariogenic and periodontal pathogenic bacteria during 20–22 h of daily aligner use may contribute to a more favorable oral microenvironment and potentially help reduce the risks of white spot lesion formation and gingival inflammation, especially when combined with routine mechanical cleaning by the patient (Al-Nadawi et al., 2021).

From a mechanistic perspective, the enhanced antibacterial effect observed in this study may be closely related to increased Cu loading and the altered surface state of PETG. Previous studies have indicated that the antibacterial action of copper and its oxides involves multiple pathways, including copper ion release, contact killing, ROS generation, and interference with bacterial membrane structure, nucleic acids, and protein metabolism, while also contributing to inhibition of bacterial biofilm formation (Ma et al., 2022; Shen et al., 2021; Pahlevanzadeh et al., 2022; Gomes et al., 2020; Salah et al., 2021). In the present study, prolonged sputtering time led to stronger Cu elemental signals and surface microtopographical changes, which was generally consistent with the observed increase in antibacterial activity. More specifically, longer sputtering times may have increased the surface Cu content, thereby providing more Cu-exposed sites at the material–bacteria interface. This may increase the local availability of Cu species and enhance Cu-mediated antibacterial activity. Cu ions may promote ROS generation and oxidative stress, thereby damaging bacterial membrane integrity and weakening bacterial viability (Gomes et al., 2020). These findings suggest that surface Cu loading and localized microstructural characteristics may jointly contribute to the antibacterial effect of the Cu-modified PETG surface. Notably, although the 45 s group exhibited more evident particulate microstructures in AFM images, quantitative roughness analysis showed that its overall nanoscale roughness remained low and did not exceed that of pristine PETG. Therefore, the reduced bacterial adhesion observed on the 45 s surface was not contradictory to its particulate morphology, because the formation of particulate Cu-related microstructures did not result in a substantial increase in overall surface roughness. Rather, the reduced bacterial adhesion may be more plausibly attributed to stronger Cu-mediated antibacterial activity associated with higher surface Cu loading, possibly involving ion-release-related effects and contact-mediated interactions. In particular, CLSM results showed reduced bacterial adhesion and weakened biofilm formation on Cu-modified surfaces, indicating that the effect of the Cu coating may not be limited to inhibition of bacterial viability, but may also involve interference with initial bacterial attachment and biofilm maturation. However, because Cu ion release, ROS levels, and bacterial membrane damage were not directly measured in this study, the proposed mechanisms remain inferential and require further experimental validation.

For oral materials, surface functionalization should not be achieved at the expense of the original material properties or biosafety. During intraoral use, clear aligners are subjected to repeated insertion and removal, occlusal contact, and friction; therefore, mechanical stability is essential for clinical application (Nugent et al., 2025). In the present study, no significant differences were observed between PETG/Cu groups and pristine PETG in terms of tensile strength, elastic modulus, maximum force, or elongation at break, indicating that Cu deposition did not markedly impair the basic mechanical properties of the PETG substrate. At the same time, the *in vitro* evaluation using HOK cells showed no significant differences in cell viability among PETG/Cu groups and the pristine PETG group at 24, 48, and 72 h. Cytoskeletal staining further showed that the cells maintained favorable adhesion and spreading, with no obvious abnormalities in actin filament organization or nuclear morphology. These findings indicate that, within the sputtering time range investigated in this study, PETG/Cu composites were able to improve antibacterial performance while preserving favorable mechanical properties and cytocompatibility, suggesting a certain balance between functionality and biosafety. This is of particular importance for the practical application of clear aligner materials.

This study still has several limitations. First, the performance of PETG/Cu composites was evaluated mainly through *in vitro* experiments, and long-term stability studies under conditions more closely resembling the oral environment are still lacking. Second, although the results demonstrated that Cu coating enhanced the antibacterial activity of the material, the specific mechanism was not further validated through Cu ion release assays, ROS measurements, or bacterial membrane damage analyses. Third, this study mainly focused on the inhibitory effects of the material on *S. mutans* and *P. gingivalis* but did not evaluate its impact on the oral commensal microbiota, nor did it directly establish enamel demineralization/remineralization models to verify its anti-caries-related potential. Future studies should incorporate ion release analysis, artificial saliva immersion, enamel demineralization models, and more complex multispecies biofilm models to more comprehensively evaluate the oral application value of PETG/Cu composites.

## 5 Conclusion

In conclusion, magnetron sputtering enabled the successful Cu functionalization of PETG surfaces and yielded PETG/Cu composites with enhanced antibacterial and antibiofilm properties. The antibacterial effects against *S. mutans* and *P. gingivalis* increased with sputtering time, while the main mechanical properties, chemical structure, and *in vitro* cytocompatibility of PETG were largely preserved. These results indicate that Cu-functionalized PETG has promising potential for the development of functional clear aligner materials. From a translational perspective, magnetron sputtering is a mature coating technology with controllable deposition and relatively short processing times, suggesting potential feasibility for scalable manufacturing. Nevertheless, the actual industrial and economic feasibility of this strategy still requires further validation through formal cost-effectiveness analysis and process integration studies before clinical translation.

## Data Availability

The original contributions presented in the study are included in the article/supplementary material, further inquiries can be directed to the corresponding authors.
